# Phase-Dependent Modulation of Oscillatory Phase and Synchrony by Long-Lasting Depolarizing Inputs in Central Neurons

**DOI:** 10.1523/ENEURO.0066-16.2016

**Published:** 2016-10-19

**Authors:** Satoshi Watanabe, Moritoshi Hirono

**Affiliations:** 1 Department of Bioengineering and Robotics, Graduate School of Engineering, Tohoku University, Sendai 980-8579, Japan; 2 Graduate School of Pharmaceutical Sciences, University of Tokyo, Tokyo 113-0033, Japan; 3 Graduate School of Brain Science, Doshisha University, Kyoto 610-0394, Japan

**Keywords:** neural oscillation, olfactory processing, phase–response curve, Purkinje cell, synchronization

## Abstract

Oscillatory neural activities have been implicated in various types of information processing in the CNS. The procerebral (PC) lobe of the land mollusk *Limax valentianus* shows an ongoing oscillatory local field potential (LFP). Olfactory input increases both the frequency and spatial synchrony of the LFP oscillation by a nitric oxide (NO)-mediated mechanism, but how NO modulates the activity in a specific manner has been unclear. In the present study, we used electrical stimulation and NO uncaging to systematically analyze the response of the LFP oscillation and found phase-dependent effects on phase shifting and synchrony. The neurons that presumably release NO in the PC lobe preferentially fired at phases in which NO has a synchronizing effect, suggesting that the timing of NO release is regulated to induce a stereotyped response to natural sensory stimuli. The phase–response curve (PRC) describes the timing dependence of responses of an oscillatory system to external input. PRCs are usually constructed by recording the temporal shifts of the neural activity in response to brief electrical pulses. However, NO evokes a long-lasting depolarization persisting for several cycles of oscillation. The phase–response relationship obtained by NO stimulation was approximately the integral of the PRC. A similar relationship was also shown for regular firing of mouse cerebellar Purkinje cells receiving step depolarization, suggesting the generality of the results to oscillatory neural systems with highly distinct properties. These results indicate novel dynamic effects of long-lasting inputs on network oscillation and synchrony, which are based on simple and ubiquitous mechanisms.

## Visual Abstract

**Figure:**
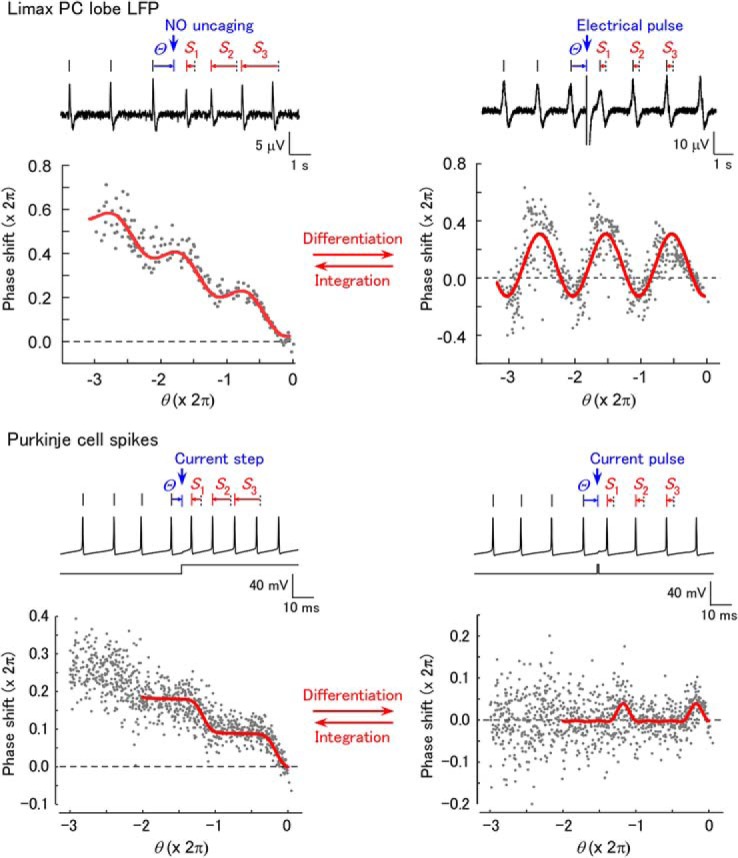

## Significance Statement

Oscillatory neural activities are modulated by sensory stimuli in a stereotyped manner, whereas isolated networks display a variety of responses to stimuli. We investigated how nitric oxide (NO)–mediated input to a molluscan olfactory center modulates the oscillatory network activity and found that its effect on network synchrony was variable depending on the stimulus phase. This suggests that the input timing should be regulated for stereotyped response to sensory stimuli, and we found that feedback inhibition of the NO-producing neurons by the rhythm-generating neurons serves to restrict the spike phase. These results suggest a novel mechanism essential for sensory processing in oscillatory networks.

## Introduction

Oscillatory activities are ubiquitous in the CNS and have been recognized as essential for sensory integration, attention, cognition, and learning (Gelperin, 2006; Wang, 2010; Buszaki, 2011; Bosman et al., 2014). Transient synchronization of oscillatory neurons has been suggested to have especially important roles in sensory processing (Gray, 1999; Frien and Eckhorn, 2000). Networks with multiple oscillatory elements often show spatiotemporal patterns of activity. Assembly of oscillators with a spatial gradient in phase exhibits repetitive wave propagation in one direction (Lam et al., 2000; Bao and Wu, 2003; Wu and Zhang, 2008; Lubenov and Siapas, 2009). Oscillatory networks can potentially exhibit a variety of responses to stimuli. For example, slices of visual cortex show planar, spiral, or irregular waves (Huang et al., 2010). However, sensory input usually evokes stereotyped responses, typically an increase in both oscillatory frequency and synchrony (Ermentrout and Kleinfeld, 2001). What cellular and network mechanisms underlie the stereotyped responses has been elusive.

Oscillatory activities have been observed in the firing of single neurons and local field potentials (LFPs). The procerebral (PC) lobe of land mollusks, which is the olfactory center essential for olfactory learning, shows a slow ongoing LFP oscillation of about 1 Hz (Watanabe et al., 2008) and has been extensively studied because of stability of the activity in semi-intact preparations and simplicity of the network structure. Olfactory stimulation increases the frequency and synchrony of the oscillation (Delaney et al., 1994) by a mechanism involving nitric oxide (NO; Watanabe et al., 2015).

One of the commonly accepted mechanisms for synchronization of a population of neurons is simultaneous input from common presynaptic neurons (Heck et al., 2007). Although this seems obvious, several points should be considered. The first point is that synaptic inputs are relatively long. For example, fast synaptic potentials mediated by AMPA receptors have a duration of tens of milliseconds, which is longer than the interval of high-frequency firing in central neurons such as cerebellar Purkinje cells. In the *Limax valentianus* PC lobe, the depolarizing effect of NO has a rapid onset but lasts several cycles of the LFP oscillation (Gelperin, 1994). This makes the input timing ambiguous. Another point is related to the timing dependence of the response of neurons. Inputs may advance or delay the subsequent oscillatory phases depending on the timing, as described by the phase–response curve (PRC). A variable amount of phase shifting in oscillating elements will result in variable effects on synchrony among those elements. This suggests the need for a mechanism to select an appropriate response from a variety of potential responses.

PRCs have been used to characterize oscillatory dynamics in a variety of neural systems (Galán et al., 2005; Gutkin et al., 2005; Lengyel et al., 2005; Tsubo et al., 2007; Stiefel et al., 2008; Phoka et al., 2010; Canavier, 2015). Using the PRC, responses of the oscillating activity to external inputs have been explained (Ermentrout, 1996; Izhikevich, 2006). The PRC is constructed by applying brief pulses at various phases of the oscillatory activity and observing how subsequent activity is temporally shifted. However, real neurons receive longer inputs, and what kind of response is evoked by long-lasting inputs is not well understood.

In the present work, we analyze the response of neural oscillations to a long-lasting input with rapid onset that continues for several cycles of oscillation, as a naturalistic approach to oscillatory dynamics. The phase–response plot after a long-lasting input appears to have a form different from that of the traditional PRC. A simple relationship between the phase–response plots after pulses and long-lasting inputs is seen in both the *Limax* PC lobe and murine Purkinje cells, which are two contrasting oscillatory systems. We also demonstrate that long-lasting inputs modulate network synchrony depending on the phase, and we reveal a possible network mechanism underlying the stereotyped response to sensory stimuli. These findings provide a basis for the understanding of oscillatory dynamics and synchronization in sensory processing, which are ubiquitous characteristics in the CNS.

## Materials and Methods

### Recording in an isolated brain preparation of Limax

An isolated brain preparation was made from *L. valentianus* from a laboratory colony (Watanabe et al., 2003). The central ganglia were placed in a recording chamber filled with saline, which contained (in mm) 70 NaCl, 2 KCl, 4.9 CaCl_2_, 4.7 MgCl_2_, 5 glucose, and 5 HEPES, pH 7.6. The LFP was recorded using a glass electrode filled with saline (tip diameter approximately 100 µm). For single-site recording, the electrode was placed near the apical end of the PC lobe (within 10% of the length of the PC lobe from the apex). For the analysis of phase lag, a second electrode was placed more basally (about 50% of the length from the apex). An AC-coupled amplifier (MEZ-2100; Nihon Kohden, Tokyo, Japan) was used to amplify the signal. The signal was bandpass filtered at 0.5–30 Hz and sampled at 1 kHz.

Perforated patch recording was made using the EPC-8 amplifier (Heka, Holliston, MA) to record the whole-cell current or membrane potential changes evoked by NO uncaging in bursting (B) neurons, as well as firing in nonbursting (NB) neurons. The electrode contained a solution of (in mm) 35 K gluconate, 35 KCl, 5 MgCl_2_, 5 HEPES, and 250 µg/ml nystatin, pH 7.2. The signals were low-pass filtered at 2 kHz and sampled at 10 kHz. To estimate the time course of the current evoked by NO uncaging in B neurons, 2 mM octanol was added to the saline to block gap junctions and suppress spontaneous activity (Ermentrout et al., 2004).

Voltage imaging of the PC lobe was made using the voltage-sensitive dye Di-4-ANEPPS (Sigma, St. Louis, MO; Kleinfeld et al., 1994; Kawahara et al., 1997). The isolated brain preparation was incubated with 86 µM Di-4-ANEPPS for 50 min and imaged using a sCMOS camera (Zyla; Andor, Belfast, UK) and an upright microscope (E-FN1; Nikon, Tokyo, Japan) with a 16× objective (NA 0.8). Images were acquired at 20 frames/s. The excitation wavelength was 517.5–542.5 nm, and the emission wavelength was >575 nm. A region of interest was set on the cell mass of the PC lobe, and the fractional change in fluorescence intensity was calculated using a custom program for MATLAB.

Electrical stimulation of the superior tentacle nerve (STN) was made using a suction electrode filled with saline. The stimuli were 3- to 5-V negative pulses with 1-ms duration, applied using an isolator (SS-403J; Nihon Kohden). To analyze the phase dependence of the response, 50–150 recordings were made at intervals of 40 s, during each of which a single stimulus was applied at a random phase of the LFP oscillation. In some experiments, NO release was blocked by incubation with 3.7 mM *N* ^ω^-nitro-l-arginine methyl ester (L-NAME; Sigma) for at least 40 min.

**Figure 1.**
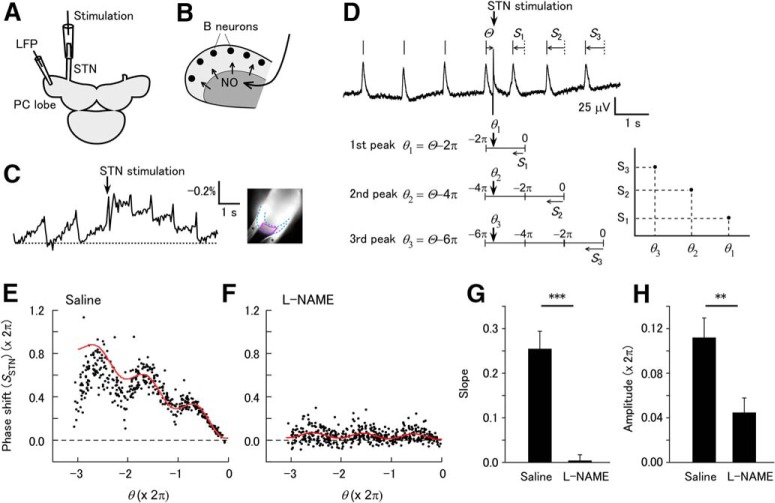
Stimulation of the STN evokes phase-dependent shifting of the LFP oscillation. (A) Schematic of the experiment. The STN was stimulated by a suction electrode. LFP was recorded from the surface of the PC lobe. (B) STN stimulation evokes NO release in the neuropil layer of the PC lobe (shaded area), which rapidly diffuses to the cell mass. The B neurons produce oscillatory activity, and NO is presumed to have uniform effects on B neurons. (C) Voltage imaging of the PC lobe reveals long-lasting depolarization after STN stimulation in the cell mass. The trace shows the fractional change of the fluorescence (negative is upward) from the cell mass. The fluorescence image of the PC lobe is shown on the right. The apical end of the PC lobe is to the bottom left. The cell mass is the area between the blue dotted curves. The red curve shows the region of interest. Two nylon threads fixing the PC lobe (asterisks) are also visible. (D) Response of the LFP oscillation to STN stimulation. The frequency of the LFP oscillation increases after STN stimulation. The LFP peaks after the stimulus shift from the times expected in the absence of the stimulus (dotted vertical lines). The phase–response plot was constructed as shown below. The phase shifts of the three peaks are denoted *S*_1_, *S*_2_, and *S*_3_. The phase of the stimulus is Θ. The relative phase θ of the stimulus is defined so the phase is zero for the unperturbed peak: $\theta_{1}=\Theta -2\pi$ for the first peak, $\theta_{2}=\Theta -4\pi$ for the second peak, and $\theta_{3}=\Theta -6\pi$ for the third peak. Finally, the phase shifts *S*_1_, *S*_2_, and *S*_3_ are plotted against the respective relative phases θ_1_, θ_2_, and θ_3_, as shown on the right. (E) Plot of the phase shift after STN stimulation [*S*_STN_(θ)] in saline. A total of 150 stimuli were applied. The red curve shows the fit with formula (2). (F) Plot of *S*_STN_(θ) in L-NAME. A total of 150 stimuli were applied. The red curve shows the fit with formula (2). (G) Slope of the linear trend [*a*_1_ in formula (2)] in saline and L-NAME. The slope was significantly larger in saline than in L-NAME (***p < 0.001, *n* = 10 for saline and *n* = 9 for L-NAME). (H) Amplitude of the sinusoidal component [*a*_2_ in formula (2)] in saline and L-NAME. The amplitude was significantly greater in saline than in L-NAME (**p < 0.01, *n* = 10 for saline and *n* = 9 for L-NAME).

For stimulation with caged NO (Gelperin, 1994), the central ganglia were incubated in saline containing 500 µM caged NO (potassium pentachloronitrosylruthenate [II]; Alfa Aesar, Haverhill, MA) for 40 min. The preparation was rinsed in saline for 10 min and placed in the recording chamber. NO was uncaged by illuminating the entire PC lobe with UV light (60- to 100-ms duration) from a 75-W xenon lamp through the epifluorescent light path of an upright microscope (BX50WI; Olympus) and an external shutter unit (OSP-EXA; Olympus, Tokyo, Japan), a filter set (U-MWU; Olympus; excitation wavelength 330–385 nm), and a 20× water-immersion objective (UMPlanFl20×, NA 0.5). ND filters were inserted in the light path to adjust the LFP frequency increase to the same level as for olfactory stimulation (Watanabe et al., 2015) and STN stimulation (Fig. 1). NO uncaging was repeated 50–150 times at 40-s intervals.

For electrical stimulation of the PC lobe, a glass suction electrode with a large tip diameter (about 200 µm) was used. The stimuli were 3- to 6-V negative pulses with 1-ms duration. To block NO release by the stimulation, L-NAME was added in the saline. The stimuli were repeated 50–200 times at 40-s intervals.

### Whole-cell recording in mouse cerebellar Purkinje cells

The experimental procedures were approved by the local committee for handling experimental animals in Doshisha University. Cerebellar slices were prepared from C57BL/6 mice of either sex at postnatal day 19–35 as described previously (Hirono et al., 2015). Parasagittal slices (250 µm thick) of the cerebellum were cut using a vibratome (VT1200S; Leica, Nussloch, Germany) in an ice-cold extracellular solution containing (in mm) 252 sucrose, 3.35 KCl, 21 NaHCO_3_, 0.6 NaH_2_PO_4_, 9.9 glucose, 0.5 CaCl_2_, and 10 MgCl_2_ and gassed with a mixture of 95% O_2_ and 5% CO_2_ (pH 7.4). The slices were maintained at room temperature for at least 1 h in a holding chamber, where they were submerged in the artificial CSF containing (in mm) 138.6 NaCl, 3.35 KCl, 21 NaHCO_3_, 0.6 NaH_2_PO_4_, 9.9 glucose, 2 CaCl_2_, and 1 MgCl_2_ (bubbled with 95% O_2_ and 5% CO_2_ to maintain pH 7.4). Individual slices were transferred to a recording chamber attached to the stage of a microscope (BX51WI; Olympus) and superfused with oxygenated artificial CSF. Purkinje cells were visually identified under Nomarski optics with a 60× water-immersion objective (NA 0.90). After establishing the whole-cell patch-clamp, spontaneous action potentials of Purkinje cells were recorded with the whole-cell current-clamp mode using MultiClamp 700B (Molecular Devices, Palo Alto, CA). Patch pipettes (2–4 MΩ) were filled with the internal solution containing (in mm) 120 K gluconate, 9 KCl, 10 KOH, 10.0 Na-HEPES, 4 NaCl, 17.5 sucrose, 10 phosphocreatine, 3 Mg-ATP, and 0.4 Na-GTP (pH 7.4). The external solution contained 100 µM picrotoxin, and the bath solution was kept at 30–31°C. The signals were low-pass filtered at 10 kHz and sampled at 20 kHz. A depolarizing current (0–100 pA) was injected to keep the baseline firing rate at 60–100 Hz. Current steps of 100 ms or pulses of 1-ms duration (amplitude 50–100 pA) were repeated 100–400 times at an interval of 0.5–2 s.

### Calculation of the phase–response relationship

The LFP in the *Limax* PC lobe and spikes of Purkinje cells were analyzed using a custom program for MATLAB (MathWorks, Natick, MA). The phase was defined as the time from the peaks of the LFP or spikes divided by the cycle period and multiplied by 2π (i.e., the peaks have phase 0 and the center points between the peaks have phase π). The absolute phase Θ of the stimulus was defined as

$$\Theta =2\pi \left(t_{\text{s}}-t_{0}\right)/T$$

where *t*_s_ is the time of the stimulus, *t*_0_ is the time of the peak just before the stimulus, and *T* is the average cycle period (average of three cycles) before the stimulus. The phase shifting in the subsequent peaks was analyzed by a method similar to the traditional PRC. However, we extended the phase–response analysis to three cycles of oscillations after the stimuli, as opposed to just one in the traditional method, to better fit the data. This was required to isolate the linear trend and periodic components that arise as a consequence of long-lasting inputs. The phase shift for the first peak after the stimulus was given by

$$S_{1}=2\pi \left(t_{0}+T-t_{1}\right)/T$$

where *t*_1_ is the time of the first peak after the stimulus. The phase shifts for the second and third peaks after the stimulus were given by

$$S_{2}=2\pi \left(t_{0}+2T-t_{2}\right)/T$$

and

$$S_{3}=2\pi \left(t_{0}+3T-t_{3}\right)/T$$

respectively, where *t*_2_ and *t*_3_ are the times of the second and third peaks. Because the phase shifts of the second and third peaks are the consequence of the stimuli applied one and two cycles earlier, the stimulus phases for the second and third peaks were considered as smaller by 2π and 4π than for the first peak. To treat the stimulus phases consistently, the relative stimulus phases for the first, second, and third peaks were defined as $\theta_{1}=\Theta -2\pi$, $\theta_{2}=\Theta -4\pi$, and $\theta_{3}=\Theta -6\pi$; by this definition, the relative stimulus phase that coincides with the peak is 0, and that of the stimuli applied at other timings has negative values. The phase shifts were plotted against the relative stimulus phases as *S*_1_ vs. θ_1_, S_2_ vs. θ_2_, and S_3_ vs. θ_3_ on the same axes (Fig. 1D). The assembly of the data obtained by repeated stimuli forms a continuous curve, usually involving a periodic component representing phase-dependent effects and a linear trend. θ_1_, θ_2_, and θ_3_ were collectively denoted as θ, and *S*_1_, *S*_2_, and *S*_3_ were denoted as *S*, with a subscript representing the type of stimulus.

The phase lag was calculated from the difference between the peak times of the LFP events recorded at the apical and basal sites. The phase lag was measured for three LFP peaks after the stimulus and was normalized by the average of the phase lag for three LFP cycles before the stimulus. The normalized phase lag was plotted against *θ* at the apical site, in the same way as the phase shift.

### Data fitting and statistical analysis

**Figure 2.**
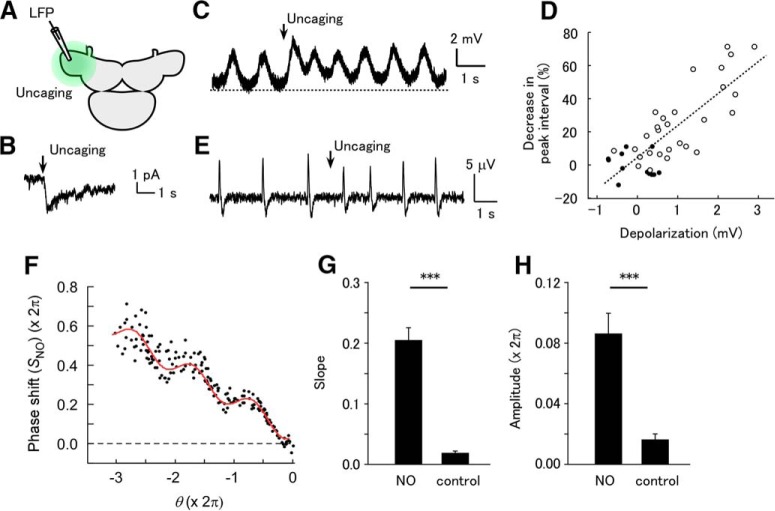
Uncaging of NO evokes phase-dependent shifting of the LFP. (A) Schematic of the experiment. NO was uncaged in the entire PC lobe by brief UV irradiation. (B) Voltage-clamp recording in a B neuron in the presence of octanol, showing NO uncaging-evoked inward current (holding potential –60 mV). (C) Current-clamp recording in a B neuron in normal saline, showing NO uncaging-evoked slow depolarization and increased frequency of periodic depolarizing events. (D) Plot of the decrease in the interval of periodic depolarizations against the membrane potential at the bottom of the interval in the neuron shown in C. Filled circles are for the intervals before NO uncaging and open circles are for the intervals after NO uncaging. The correlation coefficient was 0.795. (E) Response of LFP oscillation to NO uncaging. (F) Plot of the phase shift following NO uncaging [*S*_NO_(θ)]. A total of 60 stimuli were applied. The red curve shows the fit with formula (2). (G) Slope of the linear trend [*a*_1_ in formula (2)] in samples stained with caged NO and unstained control samples. The slope was significantly larger in stained samples (NO) than in control samples (***p < 0.001, *n* = 16 for NO and *n* = 12 for control). (H) Amplitude of the sinusoidal component [*a*_2_ in formula (2)] in stained and unstained control samples. The amplitude was significantly greater in stained samples than in control samples (***p < 0.001, *n* = 16 for NO and *n* = 12 for control).

A step input can be interpreted as a continuum of pulses. Assuming linearity, the phase shift in response to a step input is approximated by the integral of the phase–response plot with pulses. Because the NO-induced depolarization in the B neuron in the PC lobe decays relatively slowly, we used the step function assumption to fit the data. For the LFP of the *Limax* PC lobe, the traditional PRC is the plot of the phase shift in response to direct electrical pulse stimuli to the PC lobe [*S*_E_(θ); Fig. 5*C*], and this was fitted by a cosine curve of the form

(1) $$a_{0}+a_{1}\cos\left(\theta -\phi \right)$$

where ϕ is the peak phase. For STN stimulation and NO uncaging, which have a long-lasting effect, the phase–response relationships *S*_STN_(θ) (Fig. 1*D*) and *S*_NO_(θ) (Fig. 2*F*) were fitted by the integral of *S*_E_(θ) from θ to 0, which is written as

(2) $$a_{0}-a_{1}\theta -a_{2}\sin\left(\theta -\phi \right)$$

For cerebellar Purkinje cells, the traditional PRC is obtained after brief depolarizing current pulses [*S*_pulse_(θ); Fig. 6*D*] and was fitted by a cosine curve with variable peak width:

(3) $$a_{0}+a_{1}\cos\left[\theta -\phi +\gamma \sin\left(\theta -\phi \right)\right]$$

where the parameter *γ* represents the peak width. The response for the step input [*S*_step_(θ); Fig. 6*C*] is fitted by the integral of *S*_pulse_(θ) from θ to 0:

(4) $$a_{0}-a_{1}\theta +a_{2}\int_{\theta }^{0}\cos\left[\eta -\phi +\gamma \sin\left(\eta -\phi \right)\right]d\eta$$

Because the phase–response plot for Purkinje cells showed a relatively large dispersion, the plot was fitted in the range between –4π and 0. The data were fitted by the least squares method using a custom MATLAB program.

The effect of the decay time constant on phase shifting was evaluated by calculation of the peak phases in the convolution of the PRC [formula (1), with *a*_0_ = 0 and ϕ = π] and an exponentially decaying input with normalized decay time constant *λ*:

(5) $$\int_{\theta }^{0}\cos\left(\eta -\phi \right)\exp\left[-\left(\eta -\theta \right)/\lambda \right]d\eta .$$

This was calculated for the peak after the onset of the input, and the difference in the calculated peak phase from that with pulse stimuli (PRC, *λ* = 0) is plotted (Fig. 5*F*). With a step function input (*λ* = ∞), the difference in the peak phase will be π/2.

For statistical analysis of circular data, Igor Pro (WaveMetrics, Portland, OR) was used. For the test of nonuniformity in the distribution of the phase, the Rayleigh test was used. For comparison of the mean phases of two independent groups, the Watson–Williams (parametric) test was used. For comparison of two related phases, the two-sample parametric test was used. For comparison of noncircular data from two independent groups, the unpaired *t* test was used. The error bars in the figures represent the SEM (circular SEM for the circular data).

## Results

### Phase-dependent effects of NO-mediated long-lasting depolarizations

The LFP recorded from the *Limax* PC lobe exhibited a periodic oscillation. In the isolated brain preparation, the STN was stimulated through a suction electrode (Fig. 1*A*). This evokes a single-action potential in NB neurons in the PC lobe, which presumably induces NO release as seen for olfactory stimulation (Watanabe et al., 2015). From the morphology of the PC lobe and distribution of NADPH diaphorase activity (Matsuo and Ito, 2009), STN stimulation presumably induces uniform NO release in the PC lobe (Fig. 1*B*). To characterize effects of STN stimulation on the membrane potential of B neurons, voltage imaging was made in the PC lobe. The cell mass of the PC lobe shows periodic changes in the fluorescence of the voltage-sensitive dye, and this corresponds to periodic changes in the membrane potential synchronized with the LFP oscillation (Delaney et al., 1994). The optical signal from the PC lobe includes both B and NB neuron components (Kleinfeld et al., 1994), and since B neurons project in the cell mass while NB neurons project in other layers (Watanabe et al., 1998), a large fraction of the optical signal from the cell mass is expected to reflect the membrane potential of B neurons. STN stimulation evoked a long-lasting depolarization with a peak amplitude of 0.247 ± 0.043% (*n* = 6; Fig. 1*C*). The decay time constant of the depolarization was 3.93 ± 0.77 s (*n* = 6). STN stimulation also increased the frequency of the periodic depolarizations, and the frequency gradually declined during the decay phase.

STN stimulation increased the LFP frequency for several seconds and advanced the LFP peaks after the stimulus from the times expected in the absence of the stimulus (Fig. 1*D*). The frequency increase by STN stimulation was 38.0 ± 8.4% (*n* = 10), and this was similar to olfactory stimulation (about 40%, Watanabe et al., 2015). To reveal the phase dependence of the effect, STN stimulation was repeated at various phases, and phase shifting was analyzed for the three LFP peaks after the stimulus. The phase–response plot thus obtained [*S*_STN_(θ)] was the sum of a line with negative slope and a periodic curve with period 2π (Fig. 1*E*).

The negative slope indicates a steplike increase in the oscillation frequency following stimulation, which shifts the peaks after the stimulus by an amount proportional to the time after the stimulus. The point where the curve crosses the horizontal axis represents the negative of the latency of the effect in units of phase. This point was close to zero, which indicates that the latency is short compared to the cycle period of the LFP oscillation. Fitting of the plot revealed a nearly constant phase [ϕ in formula (2)] among the samples (3.90 ± 0.28 rad, circular mean ± SEM), which was significantly nonuniformly distributed (Rayleigh test, *p* = 0.030 [Table 1, line a], *n* = 10).

**Table 1.** Statistical analyses

Line	Data structure	Type of test	Power
a	von Mises distribution	Rayleigh test	NA
b	Normal distribution	Unpaired *t* test	1.000
c	Normal distribution	Unpaired *t* test	0.790
d	von Mises distribution	Rayleigh test	NA
e	Normal distribution	Unpaired *t* test	1.000
f	Normal distribution	Unpaired *t* test	0.995
g	Normal distribution	Unpaired *t* test	0.928
h	Normal distribution	Unpaired *t* test	0.573
i	Normal distribution	Unpaired *t* test	0.742
j	Normal distribution	Unpaired *t* test	0.990
k	von Mises distribution	Rayleigh test	NA
l	von Mises distribution	Rayleigh test	NA
m	von Mises distribution	Unpaired two-sample (Watson–Williams) test	0.169
n	Normal distribution	Unpaired *t* test	0.158
o	von Mises distribution	Rayleigh test	NA
p	von Mises distribution	Rayleigh test	NA
q	von Mises distribution	Paired two-sample test	0.720
r	Normal distribution	Paired *t* test	0.051

When L-NAME was added to the bath solution, both the linear and periodic components in the phase–response plot disappeared (Fig. 1*F*). Both the slope of the linear trend and the amplitude of the periodic component were decreased by L-NAME (Fig. 1*G*, *H*; slope for saline, 0.254 ± 0.04, mean ± SEM; slope for L-NAME, 0.004 ± 0.013; unpaired *t* test, *p* = 0.00010 [Table 1, line b]; amplitude for saline, 0.701 ± 0.114 rad; amplitude for L-NAME, 0.279 ± 0.086 rad; unpaired *t* test, *p* = 0.0091 [Table 1, line c]; *n* = 10 for saline and *n* = 9 for L-NAME). This suggests that NO released by STN stimulation not only increased the frequency of the LFP oscillation, but also had a phase-dependent effect that is sensitive to stimulus timing on a subsecond time scale.

Although the effects of STN stimulation suggest phase-dependent action of NO, there is also the possibility that STN stimulation triggers NO release through a phase-dependent mechanism while the action of NO is not phase-dependent. Therefore, we used NO uncaging to activate B neurons independently of the activity of NB neurons and the process of NO release (Fig. 2*A*). Voltage-clamp recording in a B neuron in the presence of octanol revealed that NO uncaging by brief UV illumination evoked a long inward current with a fast onset, which gradually decayed (Fig. 2*B*). The peak amplitude of the current was 2.71 ± 0.48 pA (*n* = 5). The rise time of the current (time from 20 to 80% of the peak) was 165 ± 28 ms (*n* = 5), and the decay time was 2.53 ± 0.47 s (*n* = 5). Under the current-clamp mode in normal saline, B neurons showed periodic depolarizations, which previous work showed are synchronized with the LFP oscillation (Kleinfeld et al., 1994). NO uncaging depolarized the membrane potential (measured at the bottom between the periodic depolarizations) by 2.56 ± 1.00 mV (*n* = 5; Fig. 2*C*). The NO-induced depolarization decayed with a time constant of 4.43 ± 1.42 s (*n* = 5). NO uncaging increased the frequency of the periodic depolarizations, and the frequency gradually declined during the decay phase. The plot of the peak interval against the membrane depolarization showed a clear correlation (Fig. 2*D*). The slope of the plot was 25.0 ± 10.0% per mV (*n* = 5), which shows how the membrane depolarization by NO is translated to the shift in the LFP timing.

Uncaging of NO in the entire PC lobe increased the frequency of the LFP oscillation for a few seconds (Fig. 2*E*). NO uncaging evoked a frequency increase (27.1 ± 3.2%, *n* = 16) that was similar to the effects of intrinsic NO released by STN stimulation (Fig. 1), indicating that NO uncaging evokes responses in the physiologically relevant range. The phase–response plot with NO uncaging, *S*_NO_(θ), had a linear trend of negative slope and a periodic component (Fig. 2*F*). Fitting of the plot revealed a nearly constant phase [ϕ in formula (2)] among the samples, which was significantly nonuniformly distributed (3.24 ± 0.18 rad; Rayleigh test, *p* = 3.5 × 10^−8^ [Table 1, line d], *n* = 16). The slope of the linear trend and the amplitude of the periodic component in the preparations loaded with caged NO were significantly larger than in the control preparations not loaded with caged NO (Fig. 2*G*, *H*; slope for the caged NO group, 0.206 ± 0.020; slope for the control group, 0.019 ± 0.003; unpaired *t* test, *p* = 1.1 × 10^−7^ [Table 1, line e]; amplitude for the caged NO group, 0.540 ± 0.082 rad; amplitude for the control group, 0.101 ± 0.025 rad; *p* = 9.15 × 10^−5^ [Table 1, line f]; *n* = 16 for NO and *n* = 12 for control). This suggests that the action of NO on the oscillatory activity of B neurons is phase dependent.

### Effects of NO-mediated inputs on network synchrony

**Figure 3.**
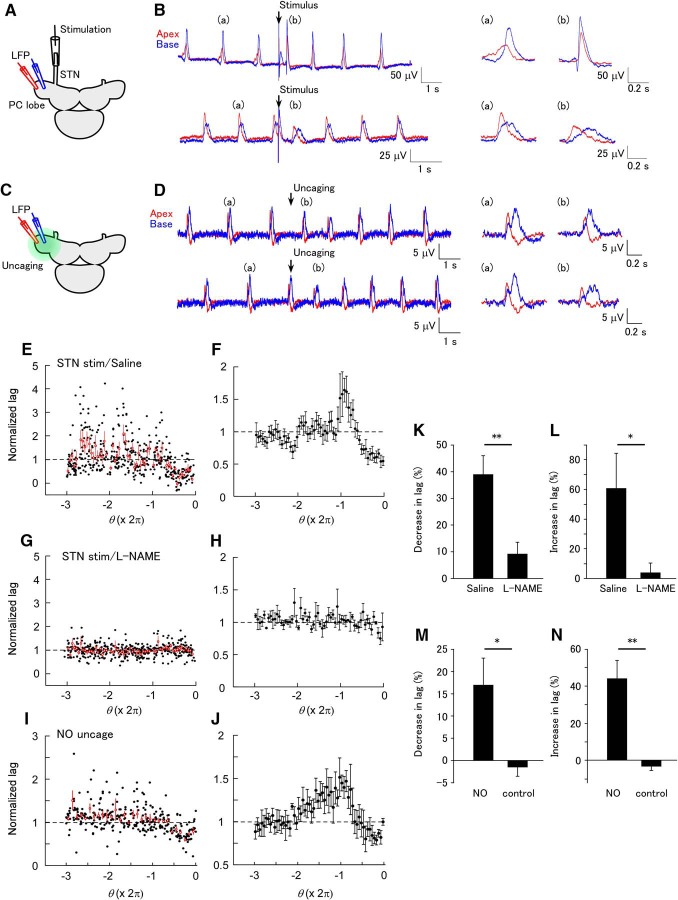
Phase-dependent modification of the spatial synchrony of the LFP by STN stimulation and NO uncaging. (A) Schematic of the experiment of STN stimulation. The LFP was recorded at apical (red) and basal (blue) sites on the PC lobe. (B) An example of the LFP showing modification of synchrony after STN stimulation. In the upper part, the STN was stimulated at a late phase in the LFP interval (Θ = 3.541 rad). Expanded LFP events before (a) and after (b) STN stimulation are shown on the right. STN stimulation decreased the phase lag. In the lower part, the STN was stimulated at an early phase in the LFP interval (Θ = 0.767 rad). STN stimulation increased the phase lag. (C) Schematic of the experiment of NO uncaging. NO was uncaged over the entire PC lobe. (D) An example of the LFP showing modification of synchrony by NO uncaging. In the upper part, NO was uncaged at a late phase in the LFP interval (Θ = 3.164 rad). Expanded LFP events before (a) and after (b) uncaging are shown on the right. NO uncaging decreased the phase lag. In the lower part, NO was uncaged at an early phase in the LFP interval (Θ = 0.302 rad). NO uncaging increased the phase lag. (E) Normalized phase lag after STN stimulation plotted against the phase of STN stimulation in normal saline. A total of 142 stimuli were applied. The average and SEM for the data in each of the bins of a size of 0.1π are shown by the red symbols. (F) Averaged plot of the normalized phase lag after STN stimulation in normal saline (*n* = 6). (G) Normalized phase lag recorded in L-NAME. A total of 145 stimuli were applied. The average and SEM for the data in each of the bins are shown by the red symbols. (H) Averaged plot of the normalized phase lag after STN stimulation in normal L-NAME (*n* = 6). (I) Normalized phase lag after NO uncaging. The average and SEM for the data in each of the bins of a size of 0.1π are shown by the red symbols. (J) Averaged plot of the normalized phase lag after NO uncaging (*n* = 6). (K) The decrease in the normalized phase lag by STN stimulation (average between –0.6π and –0.1π) in normal saline and L-NAME. The decrease was significantly larger in normal saline than in L-NAME (**p < 0.01, *n* = 10 for saline and *n* = 9 for L-NAME). (L) The increase in the normalized lag (average between –2.1π and –1.7π) in normal saline and L-NAME. The increase was significantly larger in normal saline than in L-NAME (*p < 0.05, *n* = 10 for saline and *n* = 9 for L-NAME). (M) The decrease in the normalized lag (average between –0.6π and –0.1π) by UV illumination in samples stained with caged NO and unstained control samples. The decrease in the phase lag was significantly larger in stained samples than in control samples (*p < 0.05, *n* = 6 for NO and *n* = 6 for control). (N) The increase in the normalized lag (average between –2.2π and –1.7π) by UV illumination in stained and unstained control samples. The increase in the phase lag was significantly larger in stained samples than in control samples (**p < 0.01, *n* = 6 for NO and *n* = 6 for control).

The LFP oscillation in the PC lobe has a phase lag along the apex to base axis. Each part of the PC lobe has a self-oscillating property (Ermentrout et al., 1998). We made a dual LFP recording and examined the effects of STN stimulation or NO uncaging on the phase lag (Fig. 3*A*, *C*). The phase lag decreased in response to STN stimulation applied just before the LFP peak (phase > –π), and increased in response to STN stimulation applied around the previous LFP peak (phase ≈ –2π; Fig. 3*B*, *E*, *F*). In the presence of L-NAME, most of the changes in the phase lag disappeared (Fig. 3*G*, *H*). L-NAME blocked both the decrease in the phase lag (average between –0.6π and –0.1π; Fig. 3*K*; saline, 39.0 ± 6.9%; L-NAME, 9.2 ± 4.3%; unpaired *t* test, *p* = 0.0023 [Table 1, line g]; *n* = 10 for saline and *n* = 9 for L-NAME) and the increase in the phase lag (average between –2.1π and –1.7π; Fig. 3*L*; saline, 60.7 ± 23.5%; L-NAME 4.0 ± 6.4%; unpaired *t* test, *p* = 0.0415 [Table 1, line h]; *n* = 10 for saline and *n* = 9 for L-NAME).

The phase lag between the apical and basal sites also changed after NO uncaging in a phase-dependent manner (Fig. 3*D*, *I*, *J*). The stimulus phases that evoked the largest decrease and largest increase in the phase lag were similar to those with STN stimulation. The decrease in the phase lag (average between –0.6π and –0.1π) and the increase in the phase lag (average between –2.2π and –1.7π) in the preparations loaded with caged NO were larger than in the control preparations not loaded with caged NO (Fig. 3*M*, *N*; decrease for the caged NO group, 17.0 ± 6.1%; decrease for the control group, –1.5 ± 2.0%; unpaired *t* test, *p* = 0.027 [Table 1, line i]; increase for the caged NO group, 44.2 ± 9.7%; increase for the control group, –3.4 ± 2.0%; unpaired *t* test, *p* = 0.0040 [Table 1, line j]; *n* = 6 for NO and *n* = 6 for control). These results suggest that the changes in the synchrony after STN stimulation are mediated by the phase-dependent action of NO.

### Spike phases of putative NO-releasing neurons

**Figure 4.**
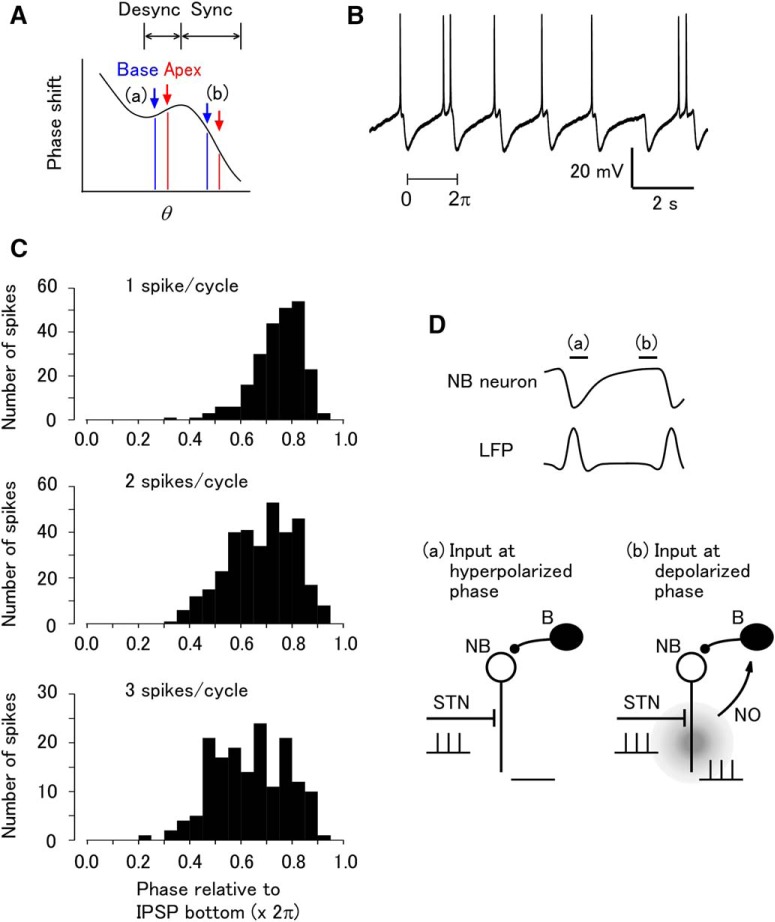
Spike phase distribution in NB neurons explains selective release of NO at the timing for synchronization. (A) The phase difference between the apical and basal oscillators leads to either synchronization or desynchronization. In the *Limax* PC lobe, the apical oscillator is advanced in phase compared to the basal oscillator. With a positive slope in the phase–response plot (a), the phase advance at the apical site is larger than at the basal site, resulting in desynchronization of the oscillation. With a negative slope in the phase–response plot (b), the phase advance at the apical site is smaller than at the basal site, resulting in synchronization. (B) Current-clamp recording in an NB neuron injected with small depolarizing DC current, showing the spontaneous spikes at late phases in the IPSP interval. (C) Spike phase distribution in NB neurons. The spike phases were grouped by the number of spikes that occurred during the cycle. (D) A possible mechanism for phase-dependent release of NO from NB neurons. NB neurons receive olfactory input from the STN, and also periodic inhibitory input from B neurons that is synchronized with the LFP. With an input at an early phase (a), the NB neuron is hyperpolarized and does not release NO (left). With an input at a late phase (b), the NB neuron fires and releases NO (right). This results in NO release only at the late (synchronizing) timing.

The apical and basal sites of the PC lobe have different phases (apical sites are more advanced in phase), and hence different amounts of phase shifting to common input at any particular instance. The different amount of phase shifting leads to either synchronization or desynchronization depending on the phase of the input (Fig. 4*A*). The NB neurons of the PC lobe presumably release NO (Matsuo and Ito, 2009), and firing of the NB neurons at some phases will decrease the phase lag, whereas at other phases it will increase the phase lag. To clarify the actual phase of firing of NB neurons, current-clamp recordings were made, and the spike phases were analyzed. Input from the STN is first transmitted to NB neurons, and then to B neurons (Inoue et al., 2000). NB neurons receive periodic inhibitory postsynaptic potentials (IPSPs) from B neurons, which are mediated by glutamate (Matsuo et al., 2009). The IPSP has been considered to be the major source of the LFP and occurs nearly synchronously with the LFP (Kleinfeld et al., 1994).

NB neurons appeared to fire preferentially at late phases in which the membrane potential is depolarized (Fig. 4*B*). A total of 42 NB neurons were analyzed, and the spikes were categorized according to the number of spikes that occurred in the IPSP interval (period between the IPSPs). When just one spike occurred in the IPSP interval, the spike phase was significantly nonuniformly distributed (Rayleigh test, 2.3 × 10^−94^ [Table 1, line k]), and the mean spike phase (relative to the IPSP troughs) was 5.05 ± 0.57 rad (circular mean ± SD, *n* = 238). This corresponds to the phase in which STN stimulation or NO uncaging was most effective at synchronizing the LFP (Fig. 3*F*, *J*). With more spikes per cycle, the range of spike timing slightly extended to an earlier phase (two spikes per interval, 4.61 ± 0.77 rad, *n* = 336; 3 spikes per interval, 4.33 ± 0.83 rad, *n* = 162), but these ranges were still in the preferred phase for LFP synchronization (Fig. 4*C*). These results suggest that the NB neurons tend to fire at a phase that matches the synchronizing timing of NO (Fig. 4*D*).

### Relationship between long-lasting and pulse inputs

**Figure 5.**
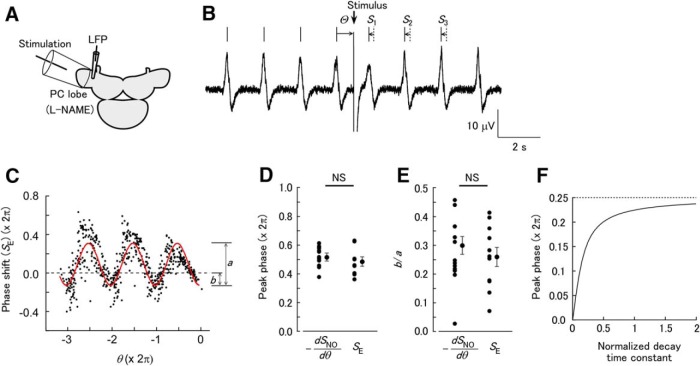
Direct electrical stimulation of the PC lobe in the presence of L-NAME evokes phase-dependent shifting of the LFP oscillation, which is equivalent to the PRC. (A) Schematic of the experiment. The apical half of the PC lobe was placed in a suction electrode for stimulation. The LFP was recorded from an electrode placed near the suction electrode. (B) Response of the LFP oscillation to stimulation of the PC lobe. After the stimulation, the LFP phase shifted. (C) Phase shift of the LFP oscillation by electrical stimulation [*S*_E_(θ)]. The red curve shows a fit with formula (1). (D) Comparison of the peak phase of –*dS*_NO_/*d*θ and *S*_E_(θ). The peak phases were not significantly different between –*dS*_NO_/*d*θ and *S*_E_ (NS, not significant; *n* = 16 for NO uncaging and *n* = 11 for electrical stimulation). (E) Comparison of the ratio of the negative component of –*dS*_NO_/*d*θ and *S*_E_ (*b*/*a* in C). The ratios were not significantly different between –*dS*_NO_/*d*θ and *S*_E_ (*n* = 16 for NO uncaging and *n* = 11 for electrical stimulation). (F) Calculated shift of the peak phase of the phase–response plot, in response to exponentially decaying inputs with different decay time constants. The abscissa is the normalized decay time constant (in units of cycle periods). The ordinate is the shift of the peak phase from that of the PRC (pulse stimuli).

The data presented above show that modulation of synchrony is dependent on the stimulus phase, and this suggests that the phase-dependent component of *S*_STN_(θ) or S_NO_(θ) is the cause of synchrony. However, the phase–response plot for the NO-mediated inputs was distinct from the traditional PRC. Therefore, we examined how the phase–response relationship for NO-mediated input is related to the PRC. To record the response to brief electrical pulses, the PC lobe was directly stimulated using a large suction electrode that covered about half of the PC lobe, whereas the LFP was recorded at a nearby position (Fig. 5*A*, *B*). To block the release of NO in response to the electrical stimulation, L-NAME was added to the saline solution. The plot of *S*_E_(θ) thus obtained had a periodic curve without a linear trend (Fig. 5*C*). The peak phase [ϕ in formula (1)] was significantly nonuniformly distributed (Rayleigh test, *p* = 1.90 × 10^−4^ [Table 1, line l], *n* = 11). To test the hypothesis that *S*_NO_(θ) has the form of the integral of the traditional PRC, two parameters were calculated for –*dS*_NO_(θ)/*d*θ and *S*_E_(θ): the peak phase and the ratio of the negative component to the amplitude. The peak phase [ϕ in formula (1)] was not significantly different for –*dS*_NO_(θ)/*d*θ and *S*_E_(θ) (Fig. 5*D*; NO uncaging, 3.24 ± 0.18 rad; electrical stimulation, 3.04 ± 0.20 rad; Watson–Williams test, *p* = 0.527 [Table 1, line m]; *n* = 16 for NO uncaging and *n* = 11 for electrical stimulation). The ratio of the negative component to the amplitude (*b*/*a* shown in Fig. 5*C*) also demonstrated no significant difference for –*dS*_NO_(θ)/*d*θ and *S*_E_(θ) (Fig. 5*E*; NO uncaging, 0.299 ± 0.031; electrical stimulation, 0.259 ± 0.033; unpaired *t* test, *p* = 0.391 [Table 1, line n]; *n* = 16 for NO uncaging and *n* = 11 for electrical stimulation). These results suggest that *S*_NO_(θ) can be explained as the integral of *S*_E_(θ), and that the phase shift in response to a continuous stimulus is approximated by the integral of the response to brief pulses. Because of gradual decay of the depolarization by NO uncaging, the depolarization is not strictly a step function. However, estimation of the peak phase of the phase–response plot with exponentially decaying inputs showed that with a decay time constant comparable to the cycle period of oscillation, the peak phase is nearly identical with that with a step function (Fig. 5*F*).

**Figure 6.**
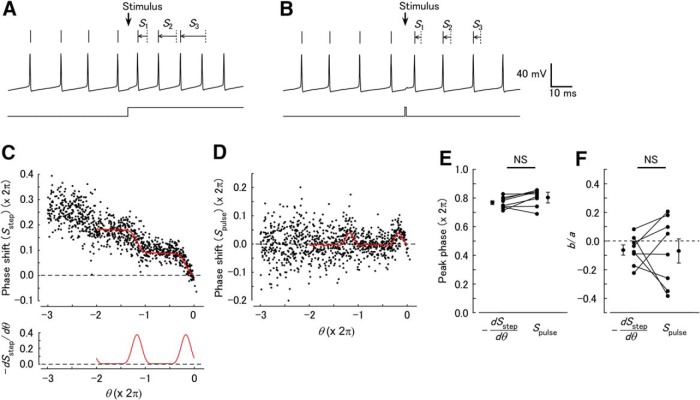
Phase-dependent shifting of spikes in mouse cerebellar Purkinje cells to step and pulse current injections. (A) Response of Purkinje cell spikes to a current step (100 pA, 100 ms). (B) Response of Purkinje cell spikes to a current pulse (100 pA, 1 ms). A and B are from the same cell. (C) Plot of phase shifting in response to step currents [*S*_step_(θ)]. The red curve shows a fit with formula (4). The differential of the fitted curve (–*dS*_step_/*d*θ) is shown below. (D) Plot of phase shifting in response to pulses [*S*_pulse_(θ)]. The red curve shows a fit with formula (3). In C and D, step and pulse stimuli (50 pA) were alternately repeated 313 times in the same cell. (E) Comparison of the peak phase of –*dS*_step_/*d*θ and *S*_pulse_(θ). The peak phases were not significantly different between –*dS*_step_/*d*θ and *S*_pulse_(θ) (NS, not significant; *n* = 8). (F) Comparison of the ratio of the negative component in –*dS*_step_/*d*θ and *S*_pulse_(θ). The ratios were not significantly different between –*dS*_step_/*d*θ and *S*_pulse_(θ) (*n* = 8).

Although the results presented above suggest phase-dependent modulation of the network activity involved in sensory processing in *Limax*, detailed mechanisms are difficult to identify, because NO induces depolarization in a number of B neurons whose characteristics have not been fully understood, and only LFP was used for the analysis of the phase–response relationship. Therefore, we also examined the phase–response relationship in the regular spiking of single cerebellar Purkinje cells and asked whether a similar relationship is obtained between long-lasting inputs and the PRC. We made current-clamp recordings in a cerebellar Purkinje cell and injected a tonic depolarizing current to induce regular 60- to 100-Hz spiking. Previous studies revealed that Purkinje cells exhibit a PRC with clear phase dependence at high firing rates (Phoka et al., 2010). Current steps (duration: 100 ms) and pulses (1 ms) were then applied alternately to construct phase–response plots. Step depolarizing stimuli of 50–100 pA increased the firing frequency of Purkinje cells by 28.5 ± 4.4% (*n* = 8; Fig. 6*A*). The frequency remained nearly constant during the stimuli. The phase–response plot [*S*_step_(θ)] had a periodic component with a negative trend (Fig. 6*C*). In contrast, pulse stimuli shifted the spike timing without a continuous change in the firing frequency (Fig. 6*B*), and the phase–response plot [*S*_pulse_(θ)] showed a periodic component without a linear trend (Fig. 6*D*). These plots were fitted by formulae (4) and (3), respectively. In contrast to the *Limax* LFP (Fig. 5), both –*dS*_step_(θ)/*d*θ (Fig. 6*C*, bottom) and *S*_pulse_(θ) had on average no negative component, which is characteristic of type 1 oscillators (Ermentrout, 1996) and consistent with a previous report (Phoka et al., 2010). For both *S*_step_(θ) and *S*_pulse_(θ), the phases [ϕ in formulae (4) and (3), respectively] were significantly nonuniformly distributed [Rayleigh test, *S*_step_(θ), *p* = 3.78 × 10^−5^ [Table 1, line o] and *S*_pulse_(θ), *p* = 1.00 × 10^−4^ [Table 1, line p]; *n* = 11). The peak phases of –*dS*_step_(θ)/*d*θ and *S*_pulse_(θ) were not significantly different [Fig. 6*E*; –*dS*_step_(θ)/*d*θ, 4.82 ± 0.08 rad; *S*_pulse_(θ), 5.05 ± 0.24 rad; paired two-sample test, *p* = 0.067 [Table 1, line q]; *n* = 8]. The ratios of the negative component of –*dS*_step_(θ)/*d*θ and *S*_pulse_(θ) were also not significantly different [Fig. 6*F*; –*dS*_step_(θ)/*d*θ, –0.064 ± 0.035; *S*_pulse_(θ), –0.069 ± 0.084; paired *t* test, *p* = 0.954 [Table 1, line r]; *n* = 8]. These results suggest that *S*_step_(θ) matches the integral of *S*_pulse_(θ).

## Discussion

### Phase-dependent effects of long-lasting depolarization on phase shifting

In the present study, we analyzed the phase–response relationships for spatially homogeneous NO-mediated input in the *Limax* PC lobe and step current input in cerebellar Purkinje cells. By extending the phase–response plot to three cycles of oscillations, linear trend and periodic components were clearly discriminated. The phase–response relationship with long-lasting inputs [*S*_STN_(θ) and *S*_NO_(θ) in *Limax* LFP and *S*_step_(θ) in cerebellar Purkinje cell spikes] consisted of a periodic component and a linear component with a negative slope, indicating a constant frequency increase and a phase-dependent effect. The analysis of the phase–response plots revealed contrasting dynamics for the two systems. Oscillatory systems are categorized into type 1 and type 2 based on the shape of the PRC (Ermentrout, 1996). In the *Limax* PC lobe, *S*_E_(θ) and –*dS*_NO_(θ)/*d*θ showed a negative component, which suggests that the LFP oscillation of the PC lobe should be categorized as type 2. Type 2 oscillators have a resonating property and are easily entrained to external input (Izhikevich, 2000, 2006). This is reasonable for the PC lobe, since it shows strong phase locking within the network. In contrast, cerebellar Purkinje cells had a type 1 PRC, because *S*_pulse_(θ) and –*dS*_step_(θ)/*d*θ demonstrated no negative component. This is consistent with previous reports that Purkinje cells have a type 1 PRC and the properties of an integrator (Phoka et al., 2010; Couto et al., 2015).

As the long-lasting function is approximated by a train of pulses, the phase shifting in response to a long-lasting function was predicted to be the integral of the phase shifting in response to pulses, as long as the linear relationship holds. In other words, the PRC is obtained by differentiating the phase–response plot by step inputs. This relationship was confirmed in both the LFP oscillation in the *Limax* PC lobe and the spikes in the cerebellar Purkinje cells, the two contrasting systems having different types of PRC (type 2 for the PC lobe and type 1 for Purkinje cells) and frequencies that differ by two orders of magnitude. In addition, the phase–response plot in Purkinje cells often exhibited a smaller variance for step stimuli than for pulse stimuli, suggesting a potential advantage of the use of step stimuli for phase–response analysis.

Another advantage of using the phase–response plot is that it enables characterization of the properties of neural transmission without observing synaptic potentials. This is advantageous when synaptic potentials are too small or difficult to isolate in the presence of spontaneous activities, or even when only field potentials can be recorded. We used the phase–response plot to evaluate fast transmission in the PC lobe and found the involvement of NO to be an essential part of the fast transmission from NB to B neurons.

The analysis of the LFP oscillation in the *Limax* PC lobe has limitations, since the response to NO may vary among the neurons constituting the network while only LFP is analyzed, and the response to NO is not a step function but decays exponentially with variable time constants. In contrast, the mechanisms underlying the dynamics of cerebellar Purkinje cells have been better studied (Fernandez et al., 2007). In both systems, however, oscillatory dynamics are generated by a number of electrical elements, and detailed quantitative data are required to reproduce the activity, which is still a challenge. On the other hand, qualitatively similar dynamics can arise from apparently distinct systems. Combining the results from the two contrasting systems will clarify essential properties of oscillatory activities, which are ubiquitous in the CNS.

### Effects of long-lasting depolarization on network synchronization

Our data suggest that modulation of spatial synchrony within the network can also be explained by the phase–response plot. STN stimulation and NO uncaging in the *Limax* PC lobe modified the spatial synchrony in a phase-dependent manner, and this presumably reflects the local phase–response relationship. The largest decrease in the phase lag corresponded to the largest negative slope in *S*_NO_(θ), and the largest increase in the phase lag corresponded to the largest positive slope in *S*_NO_(θ) (compare Figs. 1*E* and 3*F* or Figs. 2*F* and 3*J* ). These results suggest that the changes in the phase lag are explained by the spatial difference in phase shifting (Fig. 4*A*). For a negative slope, the amount of phase shift for the basal oscillator is larger than for the apical oscillator, and thus, enhances synchrony. For a positive slope, the amount of phase shift for the basal oscillator is smaller than for the apical oscillator, and thus, diminishes synchrony. Synchronization depends only on the slope of the phase–response plot, irrespective of what kind of stimuli are used. Similarly, step input presumably synchronizes Purkinje cell spikes, as judged from the phase-dependent nature of the phase–response plot.

A previous study showed that olfactory stimulation synchronizes the oscillation in the PC lobe (Delaney et al., 1994). In contrast, stimulation of the STN or NO uncaging induced both synchronization and desynchronization of the oscillation depending on the phase of the stimuli. Continuous release of NO at random phases will average out the response. This suggests that the timing of NO release should be regulated for a response in a specific direction. We found that periodic feedback inhibition of the NB neurons by B neurons restricted the timing of NO release to the preferred phase for synchronization (Fig. 4*C*). Although other mechanisms may exist for olfactory stimulus-evoked synchronization of the LFP oscillation, such as interaction between NB neurons (Ermentrout et al., 2004), the results of the present study suggest a novel simple mechanism to generate a stereotyped response in neural synchrony.

### Physiological significance of synchronization

Dynamic synchronization of neural activity is considered to be essential for sensory processing. The *Limax* PC lobe is a higher-order olfactory center, and unlike the olfactory bulb of mammals and the antennal lobe of insects, it lacks clear structural boundaries such as glomeruli. However, traveling waves along the apex-base axis seem to produce a dynamic assembly of neurons by temporally separating the activity from other neurons. The neurons clustered in a band-shaped domain, which are simultaneously activated during wave propagation, have been proposed to be memory units (Kimura et al., 1998a, 1998b; Ermentrout et al., 2001). Transient enhancement of spatial synchrony may assist interaction between the domains. The higher-order learning that *Limax* can perform requires the association of a novel stimulus with a previously learned memory (Sahley et al., 1981). Spike timing-dependent plasticity (Feldman, 2012) may help to establish the association of different units during the period of enhanced synchrony. The encoding of information in such networks can be more flexible than in structurally defined neuron groups.

Although rate coding has been considered to be the major form of information processing in the cerebellum, recent studies have also suggested the importance of spatiotemporal coding (De Zeeuw et al., 2011). Synchronized oscillation in the field potential or Purkinje cell spikes occurs mainly as a consequence of common parallel fiber input (Heck et al., 2007; Person and Raman, 2012), although direct synaptic connections between Purkinje cells also serve to synchronize spikes in juvenile animals (De Solages et al., 2008; Watt et al., 2009). Compared with the high frequency firing of Purkinje cells, fast glutamatergic synaptic potentials have a relatively long duration that may continue over the course of several spike intervals (Sakurai, 1987). The effect of synaptic input is therefore better treated as a step input than as a pulse. The results of the present study suggest that such synaptic inputs can potentially modify synchrony among Purkinje cells depending on the phase.

### Dynamic effects of NO

We revealed that NO has phase-dependent effects on the LFP oscillation of the *Limax* PC lobe. This is striking because the effects of neuromodulators are generally slow and have been considered to carry little temporal information. We suggest that the rapid onset of the action of NO is essential for the phase-dependent effects. In fact, activation of guanylyl cyclase, which mediates the main pathway of an NO-induced response, takes as little as a few milliseconds (Bellamy and Garthwaite, 2001), and NO uncaging evoked a current with an onset time constant much shorter than the cycle period for LFP oscillation (Fig. 2*B*).

NO is a highly diffusive gaseous transmitter involved in various functions in the CNS, including regulation of neurotransmission, synaptic plasticity, and neural excitability (Philippides et al., 2000; Calabrese et al., 2007; Hardingham et al., 2013). NO is also involved in precise olfactory recognition (Sakura et al., 2004) and learning (Yabumoto et al., 2008) in *Limax*. Although the involvement of NO in neural transmission in the PC lobe has been shown (Gelperin, 1994; Gelperin et al., 2000; Watanabe et al., 2015), the present results suggested that NO is essential for the dynamic effects of olfactory stimulation. We showed that L-NAME blocks—and NO uncaging mimics—the effects of STN stimulation. These data suggest that NO mediates most of the presumed fast transmission from NB neurons to B neurons, the transmitter of which has not been identified (Inoue et al., 2000).

## Synthesis

Reviewing Editor: Upinder Bhalla, National Center for Biological Sciences

Decisions are customarily a result of the Reviewing Editor and the peer reviewers coming together and discussing their recommendations until a consensus is reached. When revisions are invited, a fact-based synthesis statement explaining their decision and outlining what is needed to prepare a revision will be listed below. The following reviewer(s) agreed to reveal their identity: Ari Berkowitz. For some manuscripts transferred from JNeurosci, a decision is made to accept the manuscript without peer review and a brief statement to this effect will follow.

The reviewers both felt that the study showed clear phase-dependent

response effects in the Limax and cerebellar systems. However there were

concerns especially about the nature of the stimulus and whether

the presented data supported the interpretation in terms of responses to

step stimuli.

1. The authors should better characterize and report the neural translation of

the experimental stimulus, by way of B neuron recordings following NO uncaging.

How consistent is the time-course and amplitude? Does it correlate with the

subsequent phase-shift? How does it compare to the expected in-vivo NO

stimulus?

2. It is not clear how the olfactory pathway or NO uncaging can be treated

as step stimuli. This weakens the analysis in the paper. The authors should

better estimate the actual stimulus waveforms and use this to reanalyze the

data.

3. The authors should provide substantially more detail about the cerebellar

recordings, and relate these to their analysis of the Limax oscillator.

Detailed reviewer comments.

Reviewer 1.

This study is very helpful for understanding the role of NB neuron-released NO on oscillations and processing of olfactory signals in the Limax PC lobe. However, the generality of the findings (emphasized by the title and elsewhere) on oscillator alterations caused by multicycle, step-like stimulation is limited because their stimulation was actually not a multicycle square wave-like stimulation in Limax.

Comments to the Authors.

This study examines phase-dependent effects of brief and prolonged stimulation on the phase and frequency of oscillations in two neural oscillators, one from the Limax PC lobe and the other from mouse cerebellar Purkinje cells in slices. It argues that prolonged, step-like stimulation both increases oscillation frequency linearly, and produces phase-dependent phase shifts, in both systems, and suggests that these two effects together are a feature of multicycle, step-like stimulation of oscillators generally.

Major Comments:

On the positive side, this study shows clear phase-dependent effects on both oscillators, both on oscillation frequency and on phase shifting. The figures nicely illustrate these effects and the text is mostly clearly written. The demonstration of NO-dependent oscillator shifts in Limax is compelling.

On the negative side, the title and the main argument appear to overreach in their emphasis and generality. If one truly wanted to test whether multicycle, step-like inputs to oscillators have phase-dependent effects on oscillator frequency and phase shifting in oscillators generally, one would probably choose a system(s) (out of the many neural oscillators that have been studied in depth) in which one could deliver square-wave depolarizing inputs directly to an oscillator neuron(s), measure that input directly, and measure a component of the neural oscillator output directly to test the resulting effect. In the Limax system, by contrast, neither the experimentally delivered stimulus [which was actually a brief pulse, both for the experiments in which the STN was electrically stimulated (1 ms) and those in which NO was uncaged by a light pulse (60-100 ms)], nor the initial neural response to this stimulus was a multicycle square wave (even though the stimulation is referred to as "step", "step-like", "uniform step-like", and "a continuous stimulus" in the manuscript), and the oscillator output was monitored via an LFP electrode. In most cases, the neural translation of the experimental stimulus was not monitored, but in the one illustrated case where it was monitored (Fig. 2B), the B neuron displayed an inward current with a fast onset and a gradual decay, perhaps exponential. There is no way to know from this how much current is required to alter the oscillator frequency or phase and therefore for what period of time the oscillator was meaningfully affected by the stimulation, nor whether the varying amount of current over time would have gradually changing effects on the oscillator, which complicates interpretation of the data. Ideally, one would instead cause a square-wave inward current for multiple cycles in these neurons, but that is probably not feasible in this system. These limitations of square-wave stimulus delivery are not acknowledged or addressed in the manuscript, and they should be.

The cerebellar Purkinje cell system might be a better system to test the main hypothesis, but those experiments are described in just one paragraph and one figure without saying anything about the mechanisms producing these oscillations, so the lack of detail and emphasis makes the case less compelling for the cerebellar system than it otherwise might be; this system might warrant its own study. As it stands, the link between the two oscillators, which appear to have little in common, seems quite loose. The authors might view this as an advantage for showing the generality of their conclusions about oscillators, but this would require that both systems allow direct square-wave stimulation and direct monitoring of the oscillator outputs and that mechanisms of oscillation be understood and explained for both systems.

That said, this study is still very helpful for understanding the role of NB neuron-released NO on oscillations and processing of olfactory signals in the Limax PC lobe and could be rewritten to have that focus.

Minor Comments:

p. 4, line 9: "the slice of visual cortex shows" should be "slices of visual cortex show".

p. 5, line 25: "of LFP" should be "of the LFP oscillation".

p. 10, lines 12-13: the assertion, "Single stimulation of the STN evoked release of NO in the PC lobe (Figure 1A)" is not backed up by Fig. 1A, which is simply a diagram of the preparation, nor does it appear to be backed up by any other figure panel, as NO release was not measured.

p. 12, line 25 and elsewhere: "phase dependent" should be "phase-dependent" (as it is in most other cases) throughout the manuscript.

p. 14, line 19: it should be "plot...was", not "plot...were".

p. 19, line 21: "cvourse" should be "course".

Reviewer 2.

Comments to the Authors:

In this study phase response curves are defined for the stimulation of olfactory pathways that trigger NO release or uncaging of NO reproducing long-lasting step-depolarizations on LFP synchrony recorded in the procerebral lobe of a mollusk. The main finding is that the phase response upon step-like depolarization resembles the one of the integral of the traditional short depolarizing input.

Main criticism: The time course of NO release under natural conditions seems to be unknown. It's therefore difficult to directly compare the step-like depolarizations induced by NO uncaging with the realistic situation in vivo. Similarly, uncaging has been applied to the entire procerebral lobe which may result in a non-homogeneous onset times of the NO-mediated effects in the individual neurons of the procerebral lobe and thus may result in No-mediated conductances with different onset times in individual cells. Indeed, Figure 4 shows that there are different NO effects in dependence on the location of the recording site (apical vs basal site). How does the time course of uncaged NO compares to stimulus induced NO release? Phase response curves require the systematic change in the timing of the stimulated input as a function of the oscillating phase in order to evaluate the effect of the input on phase advance or phase delay. To my understanding this is missing in the manuscript. Only defined time points shortly before and after the oscillation are provided.
